# ‘Aquí viene una Veneca más’: Venezuelan migrants and ‘the sexual question’ in Peru

**DOI:** 10.1080/13648470.2022.2046700

**Published:** 2022-06-13

**Authors:** Rebecca Irons

**Affiliations:** University College London, Anthropology, London, UK

**Keywords:** Migration, sexual and reproductive health, HIV, Venezuela, Peru, stigma

## Abstract

Migrant access to sexual and reproductive health (SRH) services has been highlighted as an urgent priority for the 800,000+ Venezuelans who have arrived in Peru in recent years due to political and economic crisis. Venezuelan migrants in Peru, however, negotiate their access to SRH services in what anthropologists term a ‘geography of blame’, and are accused and stigmatised for having imported sexually transmitted infections to the local population. Alongside this blame, female migrants are highly sexualised and face stigma, resulting in real and perceived threats to their safety, wellbeing, and integration. By juxtaposing ethnographic research and 50 interviews conducted with female migrants living in Lima, their *Limeño* neighbours, and with local NGOs, the paper argues how stigma is itself a neglected public health issue. Addressing SRH needs for Venezuelan migrants is not *only* a question of rolling out health campaigns or providing pills, but that underlying social issues such as sexualisation and stigma need to also be recognised and incorporated into policy.

## Introduction

‘Here comes yet another *Veneca*’, a male Peruvian voice echoed from behind when Maria, a thirty-six-year-old migrant retail worker from Caracas, Venezuela, took her place in the queue for family planning at the local neighbourhood health ‘post’ in Lima Norte. This instance of a *Limeño* referring to Maria by a xenophobic slur, ‘*Veneca*’, instead of the more neutral *Venezolana*,^1^ hints at deeper issues taking place amongst the migrant community in Peru. Since the Venezuelan exodus began around the year 2015 (Armas Acosta 2019), an estimated one million plus migrants have sought refuge in Peru—where they have faced challenges in access to healthcare services. Migrant women, especially, are not only sexualised but stigmatised and accused of ‘importing’ HIV and STIs, which has contributed to concerns over physical and structural violence, and an inability to integrate and feel welcomed.

Scholars have pointed out how access to sexual and reproductive healthcare (SRH) is a particular healthcare concern for Venezuelan migrants in Peru (Mendoza and Miranda 2019). As such, it is important to address generalised perceptions that migrants bring STIs into host countries, as this may influence willingness and ability to access SRH services.

I was interested to understand these issues from the perspectives of the migrant women themselves, and to present a wider reflection on the experiences and perceptions of migrant women as ‘promiscuous importers’ of HIV and other STIs. Issues affecting migrant women, and the consequences that this may have on stigma and treatment within the host community, continue to need attention as the migrant crisis worsens.

Yet, as the migratory situation is recent and constantly evolving, we lack important data about lived experiences. This paper will address stigma and access to health care through an analysis of ‘The Sexual Question’ of racialised others in Peru (Drinot 2020), and an exploration of how stigma may be perceived as a ‘moral status’ (Kleinman and Hall-Clifford 2009). As stigma can influence the lives of those on the receiving end (ibid.), it will be important to understand the contemporary Venezuelan experience as lived in Peru. It will be concluded that, as Peru has a long history of blaming the racialised ‘other’ for importing STIs (Drinot 2020), the situation whereby Venezuelans are accused is not necessarily new nor unique. However, what is different here is that migrants genuinely need greater access to SRH (Mendoza and Miranda 2019), and HIV (and migration to search for treatments) is indeed a real problem amongst the migrant community (Cousins 2019).

Taking this ‘geography of blame’ (Farmer 2006) as a theoretical steppingstone, I argue that the social context of sexualisation and stigma need to be addressed as part of public health policy considerations. Such an approach can address the social context of needing to ‘admit’ to having SRH issues and seeking out care when holding structurally precarious positions as migrants.

## Background

### Migration and health care access

Due to the lack of access to SRH care in Venezuela, migrants arriving in Peru have particular needs that must be met. The Venezuelan health system is in a state of crisis, which, along with the economic and political emergencies that the country is now experiencing, has resulted in the mass exodus of 10% of the population (Silva-Santisteban 2019: 4).

In 2017, the Inter-American Commission on Human Rights (IACHR) reported that in Venezuela medication scarcity had risen to 90%, and 80% of hospital infrastructure was out of service (Garcia Ganoza 2019: 152). Venezuela stopped publishing health data in 2016, however analyses suggest that this situation has caused a resurgence in epidemics that had once been under control such as malaria, tuberculosis, and HIV (Page et al. 2019). Particularly, the shortage in contraceptives have induced a public health emergency, as unplanned pregnancies for migrant women experiencing insecure health care, as well as STIs, have become a major concern (Albaladejo 2018) and migrant women are at heightened risk for the consequences of unmet needs for contraception (Kessler, Goldenberg, and Quezada 2010).

Although local NGOs have attempted to fill the voids left by governmental health services, providing HIV tests, awareness campaigns, and health phone-lines (Parkin-Daniels 2018), this has hardly been sufficient to deal with the soaring deficiencies in SRH care. This situation has particularly affected those 77,000 Venezuelans living with HIV, who have been unable to access antiretrovirals (ARVs) as before, resulting in a reduction in quality of life (Garcia Ganoza 2019: 152–153). In search of proper care, around 8,000 migrants living with HIV have left the country, emigrating principally to other Latin American nations (Silva-Santisteban 2019: 4), with at least 1,700 of them using health care services in Peru in 2019^2^ (Cousins 2019: 734). In total, an estimated 800,000+ documented Venezuelans had arrived in Peru by the end of 2019, with the numbers now topping one million. However, it has been argued that the reception of Venezuelans living with HIV in Peru might have complicated the epidemic locally (ibid.), and indeed, the Venezuelan exodus has been accused of spreading infectious diseases, and HIV specifically, to the neighbouring Latin American countries of Columbia and Brazil (Rodriguez-Morales et al. 2019; Gomez 2019; Doocy et al. 2019).

While infection rates have some epidemiological truth to an extent, the media has contributed to the spread of stereotypes and myths about Venezuelan migrants importing STIs, and particularly HIV, to host countries. Online news reports referring specifically to female migrants have surfaced from Colombia and Peru, though journalists argue that this is ‘fake news’ and should be ignored (Gomez-Cruz 2019; Livise 2018). However, not everyone does ignore them. The myth that migrants bring STIs, and specifically HIV, into Peru is not only based upon a perceived ‘excess’ of untreated HIV in Venezuela. It is also based on perceptions of promiscuity and ‘immoral’ sexual behaviours within the Venezuelan population that go beyond notions of health service collapse (Jáuregui 2019).

Within Peru, HIV already carries a heavy load of stigma, however, it is largely an epidemic concentrated in the population of homosexual men and men who have sex with men (MSM) (Cousins 2019: 733). The increasing circulation of stereotypes blaming Venezuelan migrants for ‘importing’ HIV has focussed overwhelmingly on women, who host country populations perceive as potential sex workers. For example, a study by Oxfam (Rivero 2019) found that across Peru, Ecuador and Colombia, there existed the highly sexualised view of Venezuelan female migrants as prostitutes, citing ‘economic need’ and’ desperation’ as the main reasons for doing so (2019: 11–12), although the report itself suggests that these stereotypes were pre-existing and only exacerbated by the increase in migrants, rather than created by their presence (2019: 11).

When it comes to healthcare access, although Peru has offered some of free medical treatments to Venezuelan migrants, including maternal care, family planning and contraceptives, and HIV treatment, some migrants still have issues accessing the rights to health care (Garcia Ganoza 2019). Whilst preliminary quantitative data suggest that the main barriers to accessing health care are an absence of health insurance, insufficient funds, a lack of time and auto-medication (INEI 2018: 71; Hernández-Vásquez et al. 2019: 583), for SRH services specifically a key reason why women do not use the available services is an widespread information deficit, leading to unnecessary spending at private pharmacies and/or unmet contraceptive and SRH service needs (Mendoza and Miranda 2019). However, the data remains limited on this, and does not analyse women’s lived experiences of the wider social implications inherent within SRH service use, nor how this may interrelate with their perceptions of how they are treated in Peru regarding ‘The Sexual Question’, stigma, and risk.

### Histories of stigma in Peru

Importantly, the pre-existence of sexualised stereotypes towards Venezuelan migrants, and indeed wider (racialised) ‘others’ in Latin America, is nothing new.

Since colonialism, in Latin America the intersection of sex *and* race has been used as a frame within which to justify separation, judgment and division of those who exist within increasingly multicultural societies (Wade, Urrea Giraldo, and Viveros Vigoya 2008; Stolcke 2007). Indeed, there are very important differences in the ancestry and migration histories of the two countries that inform the racial identities of their residents. In contrast to Pacific-coast Peru, where 3.6% of the population identify as Afro-Peruvian (INEI 2017), Venezuela is a Caribbean country where 60% of people have some African ancestry (Brown-Vincent 2019).

Recent scholarship on the history of prostitution in Peru ties together such ideas to discuss the changing ways in which sex, sexuality, and the ‘other’ have interacted. For Drinot (2020), ‘The Sexual Question’ in Peru ‘refers to the sexual issues that stood in the way of the flourishing of the population’ and ‘the ways in which sexuality became a political issue in the nineteenth and twentieth centuries’ (2020: 7–8). Key to this framing was blaming the racialised other, with STIs viewed in ‘highly racialized ways’ (2020: 21), with blame falling to Asians, black people, the indigenous, and Jewish individuals—depending on social contexts of the time. Importantly, the racialised other(s) were portrayed as spreading disease to the more ‘desirable’ local race—white Peruvians. As such, in Peru, ‘geographies of blame’ (cf. Farmer 2006) over STIs have therefore long been racialised. The group blamed, and the group most ‘desirable’ to protect, may change depending on the social contexts of the day, but as Drinot shows, there has been a fairly consistent trajectory of STI-blame in the history of the country (and in Lima specifically).

Of course, there is nothing necessarily new or specifically Latin American in blaming a ‘foreign other’ for an STI. For example, in Europe, syphilis has been blamed on the French and Italians (Drinot 2020: 169), and the U.S. blamed Haitians for HIV. However, what is arguably specific in the present case is the interaction of sexualisation, stigma, and morality.

Kleinman and Hall-Clifford (2009) argues that stigma is as much a moral status as a psychological state. In Peru, sexualisation is deeply connected to moral status, and particularly female morality (Drinot 2020: 124). Furthermore, by denouncing the sexual immortality of the ‘other’ and stigmatising them, *Limeños* have long been able to reaffirm their own moral standing (ibid.). Stigma over sexualisation has the capacity to change social life and relationships (Kleinman and Hall-Clifford 2009), and therefore it has been argued for its inclusion in global health research with calls for a ‘new science of stigma’ (Keusch, Wilentz, and Kleinman 2006). As such, it could be suggested that sexualised stigma against Venezuelan women does more than affirm the morality of those doing the stigmatising and can lead to very real issues of violence. News reports from Peru suggest that Venezuelan women are at risk due to such sexualisation, and cases of women being drugged and raped have surfaced intermittently (La República 2019). For example, in 2017, 2,415 reports of sexual violence were reported by women in Lima (Guy 2017), and a special video news report by Peruvian network CNA in 2018 managed to capture such events as they unfolded on the streets of the capital. In the report, a young Venezuelan woman is followed by a clandestine camera crew as she sells food items on the street, and we see local Peruvian men make lewd comments about her beauty, demand that she get in their car, attempt to force their phone numbers upon her, and caress her hands when they exchange money for the purchased goods (Jenner 2018). As 46% of Venezuelans in Peru work selling items on the streets (INEI 2018), they too may be exposed to such risk, and the consequences of sexualisation and stigma may become dangerous.

Finally, it is important to note that the generalised sexualisation and stigma of Venezuelan migrants is not necessarily based on any observable facts, but instead may be a part of what Chavez (2008) calls the ‘Latino threat’, whereby migrants pose a reproductive threat to the demographics of the nation, and their sex should thereby be policed (similarly to Drinot’s conclusions for ‘The Sexual Question’). However, this should not obscure the fact that there are genuine SRH needs, as outlined above, which include the need for family planning, contraception, and HIV treatment. Furthermore, the feminisation of HIV has meant that increasing numbers of Caribbean *women* must seek treatment (Hem-Lee-Forsyth et al. 2019). This may intersect further with ‘The Sexual Question’, as in Peru concerns over STIs have long been related to discourses over the *policing of women* so as to *protect men* (Drinot 2020). Finally, as HIV is somewhat of a posterchild for sexualised stigma (Herdt 2001), this may potentially compound the problem further for migrants.

Building on these debates, I suggest that ‘The Sexual Question’, and associated racialised blaming and stigma, are not relegated to history but continue to influence perceptions of ‘the other’, and ‘the other’s’ perceptions of Peru. This time, the new ‘others’ are Venezuelans. By analysing the contemporary Venezuelan situation in Peru through ‘The Sexual Question’, it can be suggested that this is not a new phenomenon related to mass-Venezuelan migration per se but has deeper roots in the region’s history. As such, there is a strong case to suggest that stigma and sexualisation be included in health policy planning in regards to all migrants and potential ‘others’, as without this framework harmful stories of blame repeat themselves.

## Materials and methods

This study draws on qualitative data collected from July-December 2019 in Lima. 50 Venezuelan migrant women participated in semi-structured interviews that sought to find out their experiences regarding perceptions of how they are treated (in local life and the media) and how they access and perceive SRH care whilst in Peru. Women were chosen based on their being of fertile age (between 18–49) and able/needing to use SRH services. 11 had no children, with the others parenting between 1 and 4 children. Of these, 8 had left their children behind in Venezuela. 5 identified as having an STI (though HIV was only specified for 1 woman). 7 of them had been pregnant whilst in Peru (though none were pregnant at the time of the interview). Only 8 had used the Peruvian national health services (MINSA) for any health care since arriving in Peru.

Participants were recruited using a snowball method, through contacts at the United Nations Population Fund (UNFPA) Lima, who were project collaborators, the Venezuelan Union in Lima, and CCifero HIV-NGO, Lima. Recruitment sought to include participants who had been in the country for more than two months so that they had exposure to the society and health systems. Direct experience with the national health system MINSA was not a requirement, nor was active use of contraception or having children. This was to give the widest-as-possible range of perspectives on health care expectations and experience. All interviews with migrants were undertaken in Lima, in either an office space close to the Venezuelan Union, a coffee shop/café, or home, and all interviewees were living in Lima at the time of interview. Interviews were voice-recorded with consent. 2 in-depth, unstructured interviews were undertaken with NGO staff in Lima, the director of the Venezuelan Union in Peru (*Union Venezolano de Peru*), an NGO that provides principally migratory and legal advice to Venezuelan migrants in Lima (including on how to access health care), and the director of CCifero, an NGO that provides HIV-related care in Lima to migrants.

In addition to formal interviews, I lived in a high-density Venezuelan area of Lima for 6 months (Lima Norte), where I was able to carry out participant observation of the daily lives of migrants when moving around the local area (shopping, taking public transport, exercising outside, etc.), observe interactions between migrants and Peruvian locals, and informally discuss these issues with migrant (and Peruvian) acquaintances and neighbours. As an ethnographic research method when approaching health-related topics, such interactions hold far more importance than at first glance. As Rutenberg and Watkins (1997) shows, when it comes to discussion of ‘taboo’ (or stigmatising) topics such as sexual and reproductive health, there is a significant buzz *outside* the clinic. Because of this, issues relating SRH and stigma also cropped up in contexts away from official clinical and/or NGO settings, and were therefore sought to give a wider overview of the Venezuelan migrant experience.

### Ethics

Interview participants were informed about the aims of the study before participating and gave their consent. Participants are all anonymised, apart from one NGO director who gave permission for his name to be used. Interview participants were all able to give their written consent for participation. On the request of the ethics committee, no details of migratory status were asked, and participants were told at the start that they were not expected to disclose this as the admission of an illegal migratory status could compromise the researcher’s position and expose the participant. At the end of the interviews, participants were informed about free-of-charge SRH services available to them in Lima. Ethical approval was given by the University College London Research Ethics Committee.

### Data analysis

Interviews were transcribed by the researcher and coded for common themes. Where used in the text, quotes were translated by the researcher. Field notes from participant observation were coded and triangulated with interview data. Academic support for this project was provided by UNFPA Peru.

## Results

### The sexualised migrant body

Having migrated from Valencia, Venezuela a year ago, Sylvia had been working as a physiotherapist in the Northern Lima neighbourhood of Los Olivos. One day, a Peruvian male customer came into her clinic complaining of back pain. She asked him to remove his shirt and lie face down on the table as she left to fetch her assessment sheet. However, when she returned to the treatment room, she instead found him fully naked and presenting his genitals to her. Evidently, he had ‘misread’ this situation, and when Sylvia reprimanded him for lacking shame in seeking gratification of a sexual nature, he responded that he thought that was ‘what they [Venezuelans] all did’. Though her University degree in physiotherapy was proudly framed and displayed on the wall behind her, Sylvia was treated this way whilst working in a professional environment and, although traumatic for her, could report this to her boss and receive some support. For those migrant women who work on the streets selling the Venezuelan typical foods *arepas* (corn tortillas), *bombas* (doughnuts) or *tizana* (fruit punch), such sexualisation may present graver problems as they often work alone and may therefore not be able to seek help if violence is enacted towards them. For example, one woman commented that when she worked selling food goods in the street, men would look at her strangely (*raro*), and pull faces (*caras largas*), which made her feel afraid.

Perhaps unsurprisingly, when our conversations focussed on the wider question of sexualisation, Venezuelan women often expressed deep fears about what might happen to them at the hands of Peruvian men. One migrant who sold home-made arepas in the street expressed particularly strong opinions about the way the local men treated Venezuelan women, which may have been formed from her first-hand experience of selling goods to the public. Reflecting on Peruvian men, she lamented that

here [in Lima], even if you are right they will tell you that you are wrong, because there’s a difference between the ‘sexual culture’ (*la cultura sexual*) between Peru and Venezuela, here the men are slimy (*babosos*), sadistic (*sadicos*), it’s different [than Venezuela], here they will actually hit you.

Though the perception that Peruvian men might physically attack a female street-seller may seem quite extreme, other women had similarly negative views about local men, seemingly formed through news reports and understandings of Peru’s high feminicide rates as well as personal experiences and anecdotes. For example, a 25-year-old housewife commented that she had seen it in the news that ‘women get kidnapped here, like when you go to look for a job in the newspaper, my neighbour told me that they are fake adverts just so they can get you into the car and take you’. She thought that ‘Peruvian men are very jealous (*celosos*) towards Venezuelan women, they are not friendly’, and so she avoided talking to them to avoid any problems. Another woman commented that ‘here you hear so many reports about feminicide, and because of this I felt anguish (*angustia*) just for being a woman’, with a nurse also perceiving Peruvian men as ‘offensive, intolerant’ and ‘not gentlemen’. She said that Peruvian men were very ‘closed’ (*cerrados*), referring to the lack of friendliness she perceived from the local community. Whether such ideas are founded or not, the fact that sexualisation is causing women to fear work and how they may be treated by Peruvian men should be an issue of concern, particularly so if they felt it was relevant to mention in interviews relating to stigma.

Though the manifestation of sexualising stereotypes of the ‘other’ have long existed in Peru, there are potential explanations as to why Venezuelan women may be targeted and/or perceived as somehow sexually immoral and available to a Peruvian public. As the director of the Venezuelan Union in Lima stated, because of the climate and so-called ‘Caribbean culture’ in Venezuela, it is common for women to wear short skirts and shorts without this necessarily indicating anything other than comfort for the weather. However, he noted, Peruvians do not do this even in the summer, and this has led to the present situation that some may misread the intentions of migrant women. Whilst this may be so, the women interviewed also put differences in attitudes down to (culturally influenced) personality types.

For example, women expressed sentiments about Venezuelans being more ‘open’ than Peruvians, and how this could potentially have negative effects on how the host country accepts and views them. One woman said that ‘Venezuelan women are more extroverted, more open, but they [Peruvians] are so closed (*cerradas*)’. This ‘openness’ was seen by another woman in a positive light too, though the locals did not necessarily agree. She said that ‘Venezuelans are very scandalous, happy (*alegre*), and the Peruvians don’t like that’. The view of Venezuelans as ‘happier’ more generally was also shared by a retail worker, when she reflected that ‘We [Venezuelans] are very happy people (*muy alegre*)’, though she acknowledged that ‘maybe sometimes we like to drink too much!’.

These views that perceive Peruvians as more socially (and sexually) closed when compared to Venezuelan women have also been expressed as suspicions of potential jealousy, which migrant women may perceive to lead to their mistreatment by their host community. For example, some migrants held the perception that Peruvian women were jealous about Venezuelan appearances, which are globally thought to be desirable due to the number of successful ‘Miss World’ candidates produced by Venezuela, amongst other things. For example, on this topic specifically, one woman commented that: ‘Here, they [Peruvian women] are xenophobic and jealous [of Venezuelan women]’. Again, such a comment was made during an interview regarding sexualisation and stigma of migrants and how this might relate to SRH. As such, it can be understood that the migrant woman felt that the perceived xenophobia and jealousy from Peruvian women was based on a difference in sex (appeal).

When they reflect upon sexualisation and stigma, Venezuelans may also perceive that they are of a different race to Peruvians and/or that Peruvians are in fact racist people. For example, a street-seller argued that

Peruvians are not ready to receive [us], they are racist and classist. Once I called a woman here in Lima a *serrana* [person from the mountains] and she got extremely angry with me, but I didn’t mean to offend her … this is because they are the racist ones, they need to have more open minds.

One woman interpreted this difference as due to Venezuelans being ‘more used to a racial mix (*mezcla*)’, however in Peru she though it was ‘new for them and so they need more information about this (*orientarse*)’ in order to overcome their prejudices.

As such, there is the suggestion that the lack of perceived racial mixture or acceptance of difference makes Peruvians intolerant towards others (Venezuelans) who come from a more racially diverse society that does not sexualise women for their dress. Such perspectives could perhaps be accused of being rather sweeping of all Peruvians. However, migrant women’s experiences of, and concerns about, how the host community may sexualise them go beyond the notions of desire (and accompanying jealousies). As has happened previously, ‘The Sexual Question’ may also lead to a stigma of sexual disease for those ‘others’ who are seen as infected bodies coming to transmit their diseases to the local population.

#### The infected migrant body

Far from ignorant about negative perceptions of her people, 41-year-old nurse Rosa sighed about the injustice of Peruvian perceptions that made her feel stigmatised. Over a coffee she explained to me that, in Peru, ‘they have created like a myth (*un mito*) about the Venezuelan woman, that she will infect you [with an STI]’. However, this myth was damaging and not representative of all Venezuelans by any means. Rosa continued that ‘maybe that happened one time but not from the rest of us’, and that for the sake of their wellbeing ‘this myth needs to be stopped’.

One consequence of the sexualisation of migrants is that, in being viewed as promiscuous, accusations that they also are infected with STIs and HIV follow on from this, as is often a consequence of ‘The Sexual Question’. As the woman quoted above suggests, there is a ‘myth’ that Venezuelan women will infect others, and this may result in stigma against migrants, whether they have any STIs or not. This does not negate that Venezuelan women with STIs and/or living with HIV may travel to Peru, however. Problematically, if the general migrant population is sexualised and suffers from stigma, then those who genuinely are ‘infected’ cannot necessarily hope to find a welcoming community that may be understanding of their condition. To explore this ethnographically, I will discuss the case study of Reyna, a twenty-seven-year-old nurse from Maracaibo living with HIV in Lima.

Reyna had travelled to Lima specifically in search of the antiretroviral treatment that she was unable to access in Venezuela, as others may have also done. However, her migratory journey was not so simple, and nor did it start as a search for HIV-related treatment in the beginning. Her home state in Venezuela borders Colombia, and Reyna had originally crossed over with the intention of heading to Bogota to work for a few weeks and bring some money and goods back home to her family. However, whilst in Colombia she reported feeling increasingly unwell, and had to abort her plans to accumulate the essential goods and cash and return to Maracaibo. There, whilst undergoing routine tests, she received her HIV diagnosis. It came to her complete surprise as she had not been intimate with anyone since she and her husband had terminated their relationship before she left for Bogota.

Back home in Venezuela she faced the shortages of medication experienced across the country, and concluded, with the support of her family, that her only solution was to travel to Peru where they had heard she could receive free treatment. Like a majority of migrants, Reyna travelled overload for ten days before reaching the border at Tumbes, where she ‘collapsed in the street from exhaustion’ on arrival. It was more than exhaustion, though. She had developed late-stage HIV (‘AIDS’). She had been in Peru for a year by the time she and I met and reflecting back on this time she said that she thought she ‘was going to die’. ‘I was so weak that I couldn’t get out of bed for weeks, I was so skinny, like more than now, I was ready to say enough and give up’, she reflected.

Reyna had been lucky in that she was able to obtain treatment with the help of a local NGO who assisted her in obtaining state health insurance and access to free medication and care. However, this was not without incident. Whilst visiting the hospital for a fresh supply of medication, she had been approached by a Peruvian man (who also would have had HIV, by virtue of his being a patient in the same clinic) and told to ‘go back to Venezuela’ instead of seeking her treatment in ‘his country’. It may be hard to tell how this man may have identified Reyna as Venezuelan, her accent, her skin, or her clothing perhaps. However, there is also the perception that whilst HIV in Peru is concentrated in the male LGBT + community, Venezuelan women form a (more visible, at least) community.

On this matter, Julio Rondinel of Foro Salud Peru and executive director of the HIV focused NGO CCifero commented that

we have found pregnant [Venezuelan] women and women with small children, it’s not only in the LGBT community, but this also makes us think that the Venezuelan HIV community has different characteristics to the Peruvian community, which is concentrated in the LGBT community, sex workers, and trans women.

This is not to say that there are no (cis)women in Peru living with HIV, only that the Peruvian HIV community may be widely *perceived* as more male than that of Venezuela. Furthermore, and regardless of the numbers of Venezuelan women living with HIV, there is no evidence to suggest that they are engaging in sex work and/or ‘infecting’ Peruvians with HIV. Indeed, as Julio went on to explain, Venezuelan sex workers who are HIV positive but unaware of their condition and potentially spreading it within the Peruvian community (unbeknownst to them) are oftentimes male homosexual and bisexual. He explained that in Peru, three types of migrants living with HIV had arrived. The first group were ‘those who already received their diagnosis back home but were advised by their doctors to migrate in search of treatment’. This group had already arrived prepared with prior knowledge about the kind of services that they could access in the country, and how to go about receiving treatment. The second group of migrants were those who had only recently been diagnosed in Venezuela. Julio said that this group had received their diagnoses under the strained medical conditions in Venezuela, and been told that they should migrate away from the country in search of medication. The final group outlined by Julio were ‘sex workers, homosexuals, and bisexuals’. He argued that this group ‘continue to work in places where they sell sex between men’ and would have limited risk perceptions about their own HIV status. This, argued Julio, was either due to Venezuela’s collapsed health system, or their lack of access to SRH in Peru. Either way, he pointed out that the highest number of HIV-related deaths amongst Venezuelans had occurred in the ‘third’ group (sex workers, homosexuals and bisexuals), with 77 in 2018, and 50 in 2019. Due to a lack of SRH service access, Julio suggested that they may be more at risk to develop AIDS, at which point they simply ‘arrive to the hospitals to die’.

Despite Julio’s concerning comments, it would seem that HIV risk is mostly confined to sex worker communities and men who have sex with men (MSM). Therefore, it is unfounded to say that Venezuelan female migrants are ‘infecting’ Peruvians (note, that it would be unfounded to blame this solely on sex workers also). However, as with other sexualised (and racialised) stigmas, a basis in truth is not necessarily needed.

That considered, the hypothesis that migrant women might avoid MINSA SRH services for fear that they would be stigmatised as ‘infected’, may still hold some sway, as Reyna’s experience suggested. More importantly however, through exploring Reyna’s story it has been possible to highlight experiences of female Venezuelan migrant sexualisation in Peru, and perceptions of them as promiscuous and infected.

## Discussion

When it comes to ‘The Sexual Question’ in Peru (Drinot 2020), a ‘geography of blame’ (Farmer 2006) has historically existed to accuse a (foreign) racialised ‘other’ of infecting the nation with STIs and thereby ‘polluting’ the population. Through analysing the experiences of Venezuelan migrants, it can be suggested that ‘The Sexual Question’ be reconsidered as relevant for the present, too. Importantly, the concept of stigma as a moral status that can change people’s lives and community interaction in a tangible way is also necessary to bring to the table here.

Drinot’s exploration of ‘The Sexual Question’ discusses who is blamed for ‘infecting locals’ in Peru, and how this is carried out. This sexualisation of Venezuelans does more than make migrants, especially women, feel unwelcome—it also results in some women fearing Peruvians, and men specifically, because of perceived potential ‘consequences’ of this sexualisation, such as violence in the street.

When it comes to media perceptions, there are two key issues of concern here, the first being the ‘mediatization’ (Hjavard 2008) of both Venezuelan women and Peruvian men, and the second being differential constructions of femininity that this ‘mediatization’ has utilised against migrant women. To begin with the latter, interviews suggested that migrant women are potentially perceived as more ‘promiscuous’ and sexualised in Peru due to a differential presentation of self, and what participants themselves saw as their different ‘culture’ to the host country. Put simply, presentation of femininity and sexuality may be quite different in Venezuela compared to Peru, and this has arguably been utilised to stereotype and stigmatise *all* migrant women, regardless of whether they had any SRH concerns or if they dressed in a certain, supposedly more promiscuous way. Yet, even if they *were* dressing in a way considered inappropriate in Peru, it is important to note that this presentation of flesh-flashing femininity is very much tied into the Venezuelan economy and subjectivities of gender, and not something that migrants could, or should, potentially ‘change’ to be received in the host country.

For example, when discussing Venezuela’s success and fame as winners of Miss World contests, Ochoa (2014) argues that notions of performative femininity are intimately related to perceptions of subjective ‘modernity’, considered desirable in Venezuela. In adhering to a certain beauty standard, oftentimes involving ‘skimpy’ clothing, make-up, and plastic surgery, women may be responding to a specific performance of modernity that may not be expressed in the same way in other countries like Peru, where modesty may be more favourably viewed. In Venezuela, Gallegos (2016) has related this mindset to the economy, arguing that because the country has so long relied on petrol-income, with periods of boom and bust always being at the back of people’s minds, Venezuelans have become accustomed to spending their money very quickly as inflation may mean that income quickly loses value. As such, it is common to purchase branded customer goods such as clothing, which receives particular criticism in Peruvian media, as ‘poor’ migrants are chided through social media memes for owning branded sportwear. However, in Venezuela, women specifically have come to learn that investing in something permanent that cannot be affected by the economy is important: their bodies. This, suggests Gallegos, has influenced the plastic surgery industry in Venezuela.

Indeed, this approach to investing finances into one’s body is not unique to Venezuela. Edmonds (2010) has suggested that a reason impoverished Brazilians invest in plastic surgery is also as a way to ‘get ahead’ through looks. If the economy is unstable, and employment precarious, investing in body modifications and your appearance can be seen as a strategy to secure future employment. That said, this may be at odds with Peruvian understandings of femininity, and as such, may result in Venezuelan stigmatisation. In other words, ‘inappropriate’ dressing is perceived as immoral. Further, as Drinot argued, highlighting the sexual immorality of one group can be a way for *Limeños* to underscore their own, superior, morality.

Thus, it is possible that the differential approaches to outward appearances have influenced the sexualisation of Venezuelan migrant women in Peru as a moral failure, which can be considered troubling if they feel unsafe in their host country and are stigmatised because of it. It is necessary to recognise the role that the media has played in this as well, especially considering the abundance of memes that have accompanied the Venezuelan exodus, compared to any historical references to ‘The Sexual Question’ where the internet did not exist. This can be analysed through ‘Mediatization’, which sees the media as agents of cultural and social change (Hjavard 2008). In this view, media outlets do not just report on ‘facts’, but they influence them. As stereotypes about Venezuelan women as sexualised are mediatized, people may act on them (for example, see the news reports of rape and attack of migrants in Lima). This is clearly serious, though the role of the media in promoting stereotypes is also present towards the Peruvian host communities.

Note that some participants who claimed they were afraid of Peruvian men were basing this on what they had seen reported, and not necessarily their own personal experiences. This is an important point to discuss further, because not only are migrants reacting by stereotyping Peruvian men as potential rapists with ‘closed’ and undesirable personalities, but there is also a racial element to this. For example, some participants thought that Peruvians were racists and that was why they may have treated migrants badly.

As Peruvians hold stereotypes about Venezuela, so too do migrants arrive with pre-conceived ideas that may have been formed previously through media reporting. For example, when the Peruvian cholera outbreak reached Venezuela in 1991, Peru was reported as a ‘backward’ and unhygienic country (Briggs and Mantini-Briggs 2004). During the same period there was a mass migration of Peruvians due to Shining Path terrorism. At this time, those involved were negatively stereotyped as ‘Indians’, which may have influenced Venezuelan racialized perceptions of Peruvians. This all suggests that ‘The Sexual Question’ may be a contemporary regional issue of relevance, as racialised ideas about diseased others are widespread. That said, in the past, Venezuela viewed Peru as culturally ‘backward’, but not necessarily sexually so. As Farmer notes, within geographies of blame are also ‘counter-accusations’, as may be seen to an extent when Venezuelan women accuse Peruvian men of being ‘slimy’ and ‘un-gentlemanly’. This suggests that they are not ‘sexualised’ because of the way *they* present themselves, but because *Peruvian men* are the perverted ones to begin with (although no one in this study accused Peruvian men of giving migrant women HIV or another STI). Whilst counteraccusations may occur, there is nevertheless an imbalance of power—those doing the accusing (Peruvians) are not mercy to the same structural vulnerability that those being accused (Venezuelans) are. Whilst migrant women may have some agency in this regard, it is an ongoing negotiation for them.

## Conclusion

In their analysis of migrant healthcare and areas that need greater visibility and improvement, Mendoza & Miranda recommend that migrant access to SRH should become a particular priority in order to tackle the present precarity under which Venezuelans live in Peru (2019: 502). Whilst in agreement with this standpoint, this paper has suggested that intervention strategies are not only a question of rolling out health campaigns or providing pills, but that underlying social issues such as sexualisation and stigma need to also be addressed and incorporated into policy.

Keusch, Wilentz, and Kleinman (2006) suggest that stigma needs to be included in the global health agenda through the development of a ‘new science of stigma’ (2006: 523) that considers the social, economic, and personal consequences of this kind of ‘othering’. The sexualisation of Venezuelan female migrants in Peruvian and wider Latin American media, and popular discourse to an extent, can be interpreted as a stigma of ‘moral status’ (Kleinman and Hall-Clifford 2009), that has long been connected with sexuality and blaming the racialised ‘other’ of importing sexual diseases in Peru (Drinot 2020). As such, going forward it will be important to give greater attention to the way in which such concepts are constructed and contested and incorporate notions of stigma into health policy where possible. Failing to include stigma in discussions of health policy creates a breakdown in social relations between migrant women and local men. Migrants continue to fear local men, creating barriers to integration into Peruvian society. Local men maintain biases and sexualised assumptions of migrant women, informing their acts of violence.

Furthermore, such sexualised stigma is especially important when it comes to treating STIs and HIV—if Venezuelan migrant women are stigmatised *en masse* as having these diseases, then those who genuinely do need to seek treatment are placed in a complicated position. Finally, when it comes to addressing the underlying social issues that structure stigma, sexualisation, and SRH, it will be pertinent to bear in mind that sexual practices and sexuality can change as a result of host community ‘culture’, including moral behaviours and attitudes (Gonzalez-Lopez 2005).

## Notes

1 Venezuelan (female) (sp.).

2 Although this number may not be accurate as it does not account for those who are unregistered.

## References

[CIT0001] Albaladejo, A. 2018. “Contraceptive Shortages Mean Venezuela’s People Face a Sexual Health Emergency.” *BMJ* 360: 1–3. 10.1136/bmj.k1197 29572318

[CIT0002] Armas Acosta, C. 2019. “De Venezuela a la Argentina: Género, Redes y Estrategias Migratorias.” In *Después De La Llegada Realidades De La Migración Venezolana*, edited by C. Blouin. Lima: THEMIS.

[CIT0004] Briggs, C., and C. Mantini-Briggs. 2004. *Stories in the Time of Cholera: Racial Profiling during a Medical Nightmare*. California: University of California Press.

[CIT0005] Brown-Vincent, L. 2019. Afro-Venezuelan Struggles for Constitutional Recognition. Global African Worker. globalafricanworker.com. Accessed 03 January 2020.

[CIT0006] Chavez, L. 2008. *The Latino Threat: Constructing Immigrants, Citizens, and the Nation*. California: Stanford University Press.

[CIT0007] Cousins, S. 2019. “A Complex Epidemic Prevents Peru Reaching HIV Goals.” *The Lancet* 6 (11): 733–734. 10.1016/S2352-3018(19)30351-0 31672279

[CIT0008] Doocy, Shannon, Kathleen R. Page, Fernando de la Hoz, Paul Spiegel, and Chris Beyrer. 2019. “Venezuelan Migration and the Border Health Crisis in Colombia and Brazil.” *Journal on Migration and Human Security* 7 (3): 79–91. doi:10.1177/2331502419860138.

[CIT0009] Drinot, P. 2020. *The Sexual Question: A History of Prostitution in Peru, 1850s–1950s*. Cambridge: Cambridge.

[CIT0010] Edmonds, A. 2010. *Pretty Modern: Beauty, Sex, and Plastic Surgery in Brazil*. Durham, NC: Duke University.

[CIT0011] Farmer, P. 2006. *AIDS and Accusation: Haiti and the Geography of Blame*. California: University of California.

[CIT0012] Gallegos, R. 2016. *Crude Nation: How Oil Riches Ruined Venezuela*. Nebraska: Potomac.

[CIT0013] Garcia Ganoza, C. 2019. “El Derecho a la Salud de Las Personas Migrantes: Un Análisis a Partir de Los Derechos Humanos.” In *Después De La Llegada Realidades De La Migración Venezolana*, edited by C. Blouin. Lima: THEMIS.

[CIT0014] Gomez-Cruz, L. 2019. “No, Colombia No Esta En Epidemia De Sida Por Culpa De Las Venezolanas.” 31 July. Colombiacheck.Com. Accessed 02 May 2020.

[CIT0015] Gonzalez-Lopez, G. 2005. *Erotic Journeys: Mexican Immigrants and Their Sex Lives*. California: University of California.

[CIT0016] Guy, J. 2017. “Far from Home, Venezuelan Migrant Women Grapple with Harassment on Lima’s Streets.” *VOA News*. 3 October. voanews.com. Accessed 01 May 2020.

[CIT0017] Hem-Lee-Forsyth, S., O. Hunter, J. McNab, and C. Ku. 2019. “Culture and Its Impact on the Feminisation of HIV and AIDS Risk among Caribbean Women.” *International Journal of Gender and Women’s Studies* 7 (1): 17–28.

[CIT0018] Herdt, G. 2001. “Stigma and the Ethnographic Study of HIV: Problems and Prospects.” *AIDS and Behavior* 5 (2): 141–149. doi:10.1023/A:1011378811611.

[CIT0019] Hernández-Vásquez, Akram, Rodrigo Vargas-Fernández, Carlos Rojas-Roque, and Guido Bendezu-Quispe. 2019. “Factors Associated with the Non-utilization of Healthcare Services among Venezuelan Migrants in Peru.” *Revista Peruana de Medicina Experimental y Salud Publica* 36 (4): 583–591. doi:10.17843/rpmesp.2019.360.4654. 31967249

[CIT0020] Hjavard, S. 2008. “The Mediatization of Society a Theory of the Media as Agents of Social and Cultural Change.” *Nordicom Review* 29 (2): 105–134.

[CIT0022] INEI. 2017. *XII Censo de Población, VII de Vivienda y III de Comunidades Indígenas*. Lima: INEI.

[CIT0023] INEI. 2018. *Condiciones de Vida de la Población Venezolana Que Reside en Perú*. Lima: INEI.

[CIT0024] Jáuregui, A. 2019. “Mitos Y Verdades Sobre Las Personas Venezolanas Con VIH: Entrevista, and A. Julio Rondinel, Coordinador Regional De Foro salud.” *IDEHPUCP*. idehpucp.pucp.edu.pe. Accessed 01 May 2020.

[CIT0025] Jenner, F. 2018. “Venezuelan Migrants in Peru face Constant Challenges.” *Peru Reports.* 6 March. Perureports.com. Accessed 02 May 2020.

[CIT0026] Kessler, K., S. Goldenberg, and L. Quezada. 2010. “Contraceptive Use, Unmet Need for Contraception, and Unintended Pregnancy in a Context of Mexico-U.S. Migration.” *Field Action Science: Migration and Health* 4 (2): 1–6.

[CIT0027] Keusch, G., J. Wilentz, and A. Kleinman. 2006. “Stigma and Global Health: Developing a Research Agenda.” *The Lancet* 367 (9509): 525–527. doi:10.1016/S0140-6736(06)68183-X. 16473128

[CIT0028] Kleinman, A., and R. Hall-Clifford. 2009. “Stigma: A Social, Cultural, and Moral Process.” *Journal of Epidemiology and Community Health* 63 (6): 418–419. 19439576 10.1136/jech.2008.084277

[CIT0029] La República. 2019. “Churín: Extranjera Denuncia Haber Sido Violada Tras Ser Dopada Frente A Terminal Terrestre.” Larepublica.Pe. 18 November. Accessed 01 May 2020.

[CIT0030] Livise, A. 2018. “Fake News: La Noticia Sobre El 70% De venezolanas Que Llegaron A Lima Con VIH Es Falsa.” *Útero*. 26 June. utero.pe. Accessed 02 May 2020.

[CIT0031] Mendoza, W., and J. Miranda. 2019. “Venezuelan Immigration in Peru: Challenges and Opportunities from a Health Perspective.” *Revista Peruana de Medicina Experimental y Salud Publica* 36 (3): 497–503. doi:10.17843/rpmesp.2019.363.4729. 31800945

[CIT0032] Ochoa, M. 2014. *Queen for a Day: Transformistas, Beauty Queens, and the Performance of Femininity in Venezuela*. Durham, NC: Duke University.

[CIT0033] Page, Kathleen R., Shannon Doocy, Feliciano Reyna Ganteaume, Julio S. Castro, Paul Spiegel, and Chris Beyrer. 2019. “Venezuela’s Public Health Crisis: A Regional Emergency.” *The Lancet* 393 (10177): 1254–1260. doi:10.1016/S0140-6736(19)30344-7. 30871722

[CIT0034] Parkin-Daniels, J. 2018. “Accion Solidaria: Propping up HIV Treatment in Venezuela.” *The Lancet. HIV* 5 (10): e549. doi:10.1016/S2352-3018(18)30263-7. 30319121

[CIT0035] Rivero, P. 2019. *Yes, but Not Here: Perceptions of Xenophobia and Discrimination towards Venezuelan Migrants in Colombia, Ecuador and Peru*. New York: OXFAM.

[CIT0036] Rodríguez-Morales, Alfonso J., D. Katterine Bonilla-Aldana, Miguel Morales, José A. Suárez, and Ernesto Martínez-Buitrago. 2019. “Migration Crisis in Venezuela and Its Impact on HIV in Other Countries: The Case of Colombia.” *Annals of Clinical Microbiology and Antimicrobials* 18 (1): 9. doi:10.1186/s12941-019-0310-4. 30849989 PMC6407272

[CIT0037] Rutenberg, N., and S. Watkins. 1997. “The Buzz Outside the Clinics: Conversations and Contraception in Nyanza Province, Kenya.” *Studies in Family Planning* 28 (4): 290–307. doi:10.2307/2137860. 9431650

[CIT0038] Silva-Santisteban, A. 2019. “Diagnóstico Rápido: Situación De Los Migrantes Venezolanos Con VIH En El Perú.” UNAIDS. https://Data2.Unhcr.Org/En/Documents/Download/69615. Accessed 01 May 2020.

[CIT0040] Stolcke, V. 2007. *Racismo y Sexualidad en la Cuba Colonial*. Madrid: Alianza.

[CIT0043] Wade, P., F. Urrea Giraldo, and M. Viveros Vigoya. 2008. *Raza, Etnicidad y Sexualidades: Ciudadanía y Multiculturalismo en América Latina*. Bogota: CES.

